# Single-molecule force spectroscopy reveals binding and bridging dynamics of PARP1 and PARP2 at DNA double-strand breaks

**DOI:** 10.1073/pnas.2214209120

**Published:** 2023-05-22

**Authors:** Nicholas A. W. Bell, Justin E. Molloy

**Affiliations:** ^a^Single Molecule Enzymology Laboratory, The Francis Crick Institute, London NW1 1AT, United Kingdom; ^b^Department of Physics and Astronomy, University College London, London WC1E 6BT, United Kingdom; ^c^Laboratory for Molecular Cell Biology, University College London, London WC1E 6BT, United Kingdom; ^d^Centre for Mechanochemical Cell Biology, Warwick Medical School, Coventry CV4 7AL, United Kingdom

**Keywords:** DNA repair, magnetic tweezers, PARP, DNA–protein interactions

## Abstract

Poly(ADP-ribose) polymerases (PARPs) play key roles in DNA damage repair pathways in eukaryotic cells. Human PARPs 1 and 2 are catalytically activated by damage in the form of both double-strand and single-strand DNA breaks. Recent structural work indicates that PARP2 can also bridge two DNA double-strand breaks (DSBs), revealing a potential role in stabilizing broken DNA ends. In this paper, we have developed a magnetic tweezers–based assay in order to measure the mechanical stability and interaction kinetics of proteins bridging across the two ends of a DNA DSB. We find that PARP2 forms a remarkably stable mechanical link (rupture force ~85 pN) across blunt-end 5′-phosphorylated DSBs and restores torsional continuity allowing DNA supercoiling. We characterize the rupture force for different overhang types and show that PARP2 switches between bridging and end-binding modes depending on whether the break is blunt-ended or has a short 5′ or 3′ overhang. In contrast, PARP1 was not observed to form a bridging interaction across blunt or short overhang DSBs and competed away PARP2 bridge formation, indicating that it binds stably but without linking together the two broken DNA ends. Our work gives insights into the fundamental mechanisms of PARP1 and PARP2 interactions at double-strand DNA breaks and presents a unique experimental approach to studying DNA DSB repair pathways.

## Significance

PARP [Poly(ADP-ribose) polymerase] proteins are important signaling enzymes in eukaryotic DNA damage repair. We investigated how PARP1 and PARP2 bind at DNA double-strand breaks (DSBs) by developing a single-molecule magnetic tweezers assay to measure protein binding at DSBs while controlling DNA tension, supercoiling, and end-chemistry. We found that PARP2 forms an extremely stable mechanical bridge across blunt DNA ends and restores DNA torsional continuity. We also discovered that PARP2 switches between end-binding and bridging modes depending on whether overhangs have 5′ or 3′. PARP1, in contrast, binds at DSB ends but does not form a bridge when opposing ends are brought together. Our results reveal fundamental mechanisms of how PARP proteins engage at DSBs, which may aid in inhibitor development.

A typical mammalian cell undergoes tens of thousands of DNA damage events per day (1). These events include chemical modifications to DNA bases as well as single-strand breaks (SSBs) and double-strand breaks (DSBs) in the phosphodiester backbone (2). DSBs are a particularly toxic form of DNA damage since they disrupt the continuity of the genome, potentially leading to chromosomal rearrangements (3, 4). DSBs are repaired by a variety of pathways including homologous recombination, nonhomologous end joining, and microhomology-mediated end joining (5).

The human poly(ADP-ribose) polymerase (PARP) family of proteins includes PARPs 1-3 which are DNA damage response proteins that are known to bind to a variety of types of SSBs and DSBs (6, 7). Binding activates PARP catalytic activity resulting in the synthesis of poly(ADP-ribose) chains, which loosens local chromatin architecture and recruits additional repair proteins to the damage site (8). PARP inhibitors block the synthesis of poly(ADP-ribose) and cause trapping of PARP proteins at sites of DNA damage (9). Cells with deficiencies in homologous recombination, such as BRCA mutant cells, are highly sensitive to PARP inhibition (10, 11). This sensitivity has been exploited by PARP inhibitor chemotherapies which have recently reached the clinic (12).

Structural studies have given insight into the mechanisms of PARP1 and PARP2 binding to a variety of types of DNA breaks (13). Truncated forms of PARP1 in complex with an SSB or DSB have shown the critical domain contacts and how DNA binding allows Nicotinamide adenine dinucleotide (NAD^+^) access to the active site through an allosteric unfolding of regulatory alpha-helices in the catalytic domain (14–17). Recently, cryo-EM and X-ray crystallography have revealed structural information on PARP2 binding to blunt-end DSBs present on naked DNA as well as in a chromatin context where DNA was wrapped around nucleosomes (18–21). These published structures all show PARP2 forming a 2:2 bridging complex with DSBs at DNA duplex ends. In cell culture models, PARP1 and PARP2 have been implicated in a variety of roles in the choice of DSB repair pathway (22). For instance, PARP1 was observed to compete with the Ku complex for DSB binding (23, 24) and antagonize end-resection (25). In contrast, PARP2 has been found to promote end-resection by limiting 53BP1 binding, thereby disfavoring nonhomologous end joining repair (26). Overall, these studies implicate PARP1 and PARP2 as key proteins in DSB repair and motivate the creation of controlled experimental systems to further our understanding of the basic mechanism of how they bind at DSBs.

In this paper, we report a single-molecule force spectroscopy technique to measure the binding of proteins across a DNA DSB. We synthesized a DNA molecule with a polyethylene glycol (PEG)–based linker which constrains two DNA ends and used magnetic tweezers to controllably apply tension and supercoiling to the DNA molecule while measuring its extension. This method allows us to perform highly controlled experiments which directly measure the kinetics and mechanics of PARP proteins as they form a bridge across broken DNA ends. We find that PARP2 forms an extremely stable bridge across blunt DNA ends and restores DNA torsional continuity. This bridging is specific to 5′-phosphorylated ends and is not observed when dephosphorylated ends are present. We also characterize different types of overhangs and find differences in the interaction modes for 3′ and 5′ overhangs. In comparison, PARP1 did not form a bridging link for any of the DNA end chemistries tested and competed away PARP2 bridge formation. Our results elucidate the fundamental properties of PARP protein interactions at DSBs and set a force scale for PARP binding at DNA breaks.

## Results

### A Single-Molecule DNA Construct with a Constrained DSB.

**Fig. 1.**
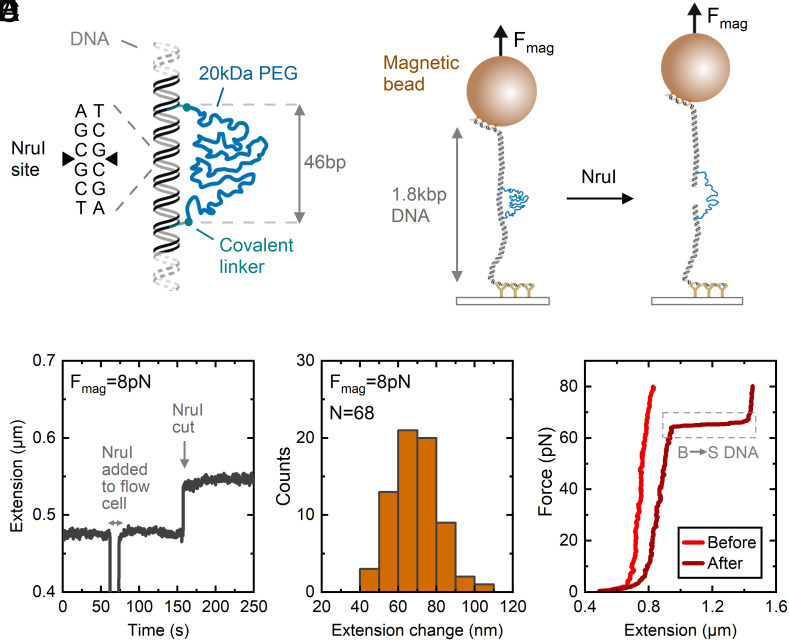
Construction of a DNA molecule for measuring bridging mechanics at DNA double-strand breaks. (*A*) Schematic of DNA molecule synthesized with a 20 kDa PEG chain linking two thymine bases using copper-free click chemistry. The PEG attachment points are separated by 46 bp along the DNA contour. The DNA sequence was designed with a recognition site at its center for NruI. (*B*) Experimental setup showing a single 1.8 kbp DNA molecule containing the PEG linker. A superparamagnetic bead is attached via multiple streptavidin–biotin attachments and surface attachments are via multiple digoxigenin–antidigoxigenin interactions. Incubation with NruI digests the DNA, leaving two blunt ends tethered together by the PEG. (*C*) Example data trace showing bead height (extension) before and after adding NruI. A constant force of 8 pN was applied throughout. A step increase in extension occurs after addition of NruI to the flow-cell, indicating successful cleavage of the DNA. (*D*) Histogram of extension change after addition of NruI using data from 68 separate magnetic beads. (*E*) Force–distance curves before and after NruI cutting. NruI cutting removes the torsional continuity of the DNA molecule, resulting in an overstretching (B–S) transition.

To study the mechanics of binding at a double-strand DNA break, we developed a method for making a double-stranded DNA molecule featuring a 20 kDa PEG molecule bridging across a central section (Fig. 1*A*). The construct was synthesized in a series of steps beginning with a copper-free click reaction between an oligonucleotide containing an internal dibenzylcyclooctyne and a homobifunctional azide–terminated PEG. This reaction creates a stable, covalent linkage between the PEG and DNA. Annealing with complementary oligonucleotides followed by a ligation reaction with PCR amplicons formed the final structure (*SI Appendix*, Figs. S1–S3). The DNA molecule is 1.8 kbp in length and is flanked by sections labeled with multiple digoxigenins and biotins for attachment to a coverslip surface and superparamagnetic bead, respectively. Light scattering measurements of PEG (27) show that the mean end-to-end distance (M_e-e_) obeys a power law dependence on molecular weight (M_w_) of M_e-e_ = 0.047*(M_w_)^0.588^, giving a value of M_e-e_ = 16 nm for M_w_ = 20 kDa (*SI Appendix*, Fig. S4). We designed the two labeled thymine bases on our synthetic DNA oligonucleotide to have a separation of 46 bp = 16 nm (given 0.34 nm/bp) in order to match the PEG mean end-to-end length. Therefore, upon formation of a DSB in between the two PEG attachment points, the PEG will hold together the two DSB ends in close proximity under conditions of low or zero force. To enable the creation of such a break, we positioned a recognition site for the restriction enzyme NruI which cuts both strands at the same position, leaving a blunt end. Fig. 1*B* shows a schematic of the DNA before and after restriction enzyme digestion showing how the PEG linker holds together the broken DNA ends. Previous single-molecule studies investigating the behavior of DNA ends under force have used a DNA-based linker, for instance, when analyzing base-stacking interactions or nonhomologous end joining kinetics (28–30). Our synthesis strategy using PEG negates any potential confounding effects due to proteins binding to a DNA linker while allowing controlled manipulation of the DNA break under force.

To test the correct assembly of the DNA construct, we tracked DNA extension as a function of time before and after adding NruI (Fig. 1*C*). A constant force of 8 pN was applied throughout. Addition of NruI results in a step change in DNA extension of 64 nm in this example trace. Fig. 1*D* shows a histogram of measurements from 68 magnetic beads, revealing a mean extension change of 69 ± 12 nm (mean ± SD). Our observed extension is consistent with a 20 kDa PEG chain (contour length = 159 nm), giving an expected extension of 56 nm at 8 pN based on a worm-like chain model (*SI Appendix*, Fig. S5). The DNA cleavage by NruI could be observed on multiple beads across the field of view—the time to cleavage varied from bead to bead due to the stochastic arrival of the enzyme to its recognition site (*SI Appendix*, Fig. S6). Fig. 1*E* shows a comparison of the force–extension curve for one bead before and after cutting by NruI. PEG has a much lower persistence length (~0.4 nm) compared to double-stranded DNA (~50 nm) and therefore it requires higher forces to stretch it to a given percentage of its contour length. This is reflected by the shallower gradient of the force–extension curve at forces <15 pN for the curve after NruI cleavage. An overstretching transition is observed after cutting, whereas no such transition is measured before cutting. Previous single-molecule force spectroscopy experiments have shown that torsionally unconstrained DNA forms an overstretched structure (a so-called B–S transition) at approximately 65 pN, whereas torsionally constrained DNA shows this transition at approximately 115 pN (31). Our measured force–extension curves are consistent with this since before cleavage, the DNA is torsionally constrained by the multiple digoxigenin and biotin bonds used for tethering, whereas after cutting, the DNA loses torsional continuity since the single C–C and C–O bonds that make up the PEG chain are able to rotate freely.

### PARP2 Forms a Highly Mechanically Stable Bridge across 5′ Phosphorylated Blunt DNA Ends.

**Fig. 2.**
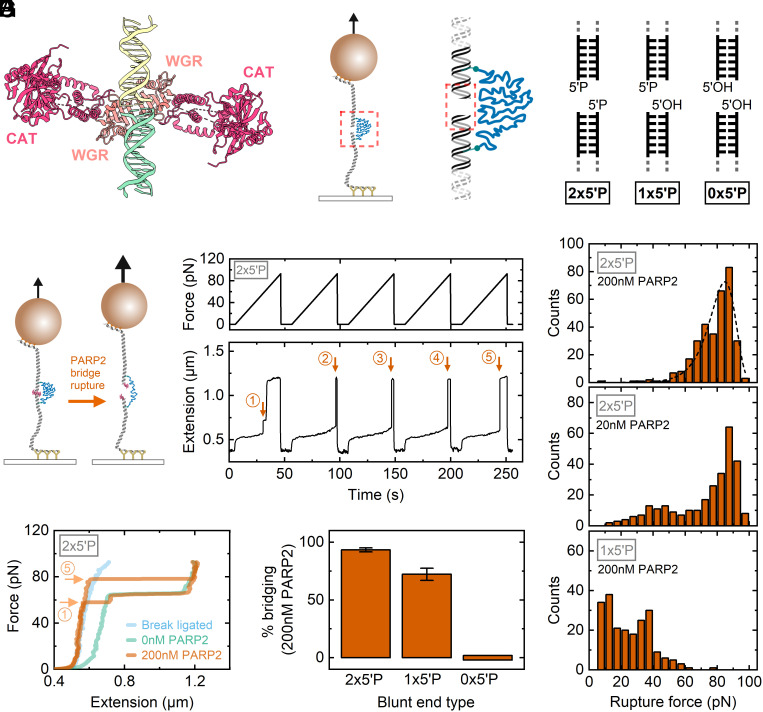
Force-induced rupturing of PARP2 at a double-strand break. (*A*) X-ray crystal structure (PDB:7aeo—ref. 19) of PARP2 bridge formed across two 5′ phosphorylated, blunt DNA duplexes. The structure shows two PARP2 proteins—the WGR and catalytic domains of each are labeled. (*B*) Schematic of magnetic tweezers showing formation of a double-strand break after incubation with NruI. Subsequently, different 5′ phosphorylated states were prepared by incubation with Antarctic phosphatase and T4 polynucleotide kinase. (*C*) Schematic of PARP2 bridge rupture under force. (*D*) Experimental trace showing successive force ramps in the presence of 200 nM PARP2. The orange arrows mark the points of rupture. (*E*) Force–extension curves comparing the traces shown in (*D*) with fully ligated DNA (before NruI cleavage–blue) and after break formation but in the absence of PARP2 (green). (*F*) Fifteen consecutive force–extension ramps were applied and the mean percentage of ramps showing a bridging event was measured. Error bars show SEM. N = 6 beads were measured for each data column. (*G*) Histograms of rupture force for 2 × 5′P and 1 × 5′P in the presence of 200 nM PARP2 and 2 × 5′P in the presence of 20 nM PARP2. The data for 2 × 5′P and 1 × 5′P at 200 nM are cumulative from N = 6 beads each and data from 20nM 2 × 5**′**P are from N = 17 beads. The dashed line shows the Bell–Evans fit for the 200 nM PARP2 and 2 × 5′P histogram (see main text for details).

Having established a method to create two opposing double-stranded DNA breaks tethered by a PEG linker, we applied our system to study the mechanical bridging of a DNA DSB by PARP2. Fig. 2*A* shows the crystal structure (PDB: 7AEO) of PARP2 in complex with DNA (19). A 2:2 stoichiometry of blunt-end, 5′ phosphorylated DNA duplexes with PARP2 was observed in this structure and also in separate literature reports which employed cryo-electron microscopy (18, 20). Each PARP2 protein forms its own bridge via its WGR domain, and there are no significant contacts between the two PARP2 proteins. Previous work suggests that PARP2 activation is highly dependent on the presence of a 5′ phosphate on both SSBs and DSBs (6, 7, 18). After NruI cleavage, the DNA is left with dual 5′ phosphorylated blunt ends. To test how the presence of 5′ phosphate affected binding, we also established methods to prepare the magnetic tweezers construct with either a single or no 5′ phosphate (Fig. 2*B*) by treating with Antarctic phosphatase and T4 polynucleotide kinase (*SI Appendix*, Figs. S7 and S8).

Fig. 2 *C* and *D* shows a schematic and example time-series data trace of an experiment to measure the rupture force of PARP2 bridging across a DSB. After addition of PARP2 to the flow-cell, the force was initially held close to 0 pN so that the two DNA ends are held in close proximity by the PEG linker, thereby allowing the PARP2 to bind across the DNA DSB interface. The force was then linearly ramped at 2 pN/s. A sudden increase in extension is observed when the PARP2 bridge ruptures as marked by the orange arrows. We found that this force-induced rupture could be repeatedly observed by returning the force to close to 0 pN—allowing the PARP2 bridge to reassemble—before ramping the force again as shown by the five consecutive force ramps displayed in Fig. 2*D*. Fig. 2*E* shows comparisons of the force–extension curves at different experimental stages, namely before NruI cleavage, after NruI cleavage, and after addition of 200 nM PARP2. In the presence of 200 nM PARP2, the curve tracks along the fully ligated DNA case before rupture at which point the curve then follows along the broken DNA case. The first force ramp shows a rupture event before the 65 pN overstretching where we observe an increase in the extension of 151 nm corresponding to the 20 kDa PEG contour length. Remarkably, as illustrated by the force–extension curve of the fifth force ramp, we observe that most ramps show rupture at a force greater than the overstretching transition of 65 pN. This indicates that PARP2 bridging restores the torsional continuity of DNA so that the DNA can support forces greater than 65 pN before the rupture of the PARP2 bridge is immediately followed by overstretching of the DNA.

As illustrated in Fig. 2*D*, we found that after force-induced rupture, the PARP2 bridge could be reformed by reducing the force close to zero so that the PEG linker then brought the two ends in proximity again. This enabled us to perform repeated force ramps to build up statistics on 1) how many cycles showed a characteristic rupture event and 2) the force at rupture. In Fig. 2*F*, we quantify the percentage of force ramps (calculated from the first 15 ramps after protein addition) that show a rupture event in the presence of 200 nM PARP2. For dual 5′ phosphate (2 × 5′P), we observe that 93 ± 4% (mean ± SEM, N = 6 beads) of force ramps show bridging. In the case of single 5′ phosphate, we observed that 72 ± 13% (mean ± SEM, N = 6 beads) of curves showed a characteristic rupture event. In the case of no 5′ phosphates on the DNA ends, we observed a complete absence of bridging. In Fig. 2*G*, we show histograms that quantify the rupture force distribution for the dual 5′ phosphate (at 200 nM and 20 nM PARP2 concentration) and single 5′ phosphate constructs (at 200 nM). At 200 nM PARP2 concentration, for the dual 5′ phosphate, we observe a single peak at approximately 85 pN. We fitted these data to the Bell–Evans model which models the rupture of single bonds under force as a lowering of the activation energy barrier by an amount *Fδ* where *F* is the force and *δ* is the distance between the bound state and activation energy peak (32, 33). At constant loading rate, the rupture force probability distribution is given by:

$$p\left(F\right)=\frac{1}{t_{on}R}\exp\left(\frac{k_{B}T}{t_{on}R\delta }\right)\exp\left(\frac{F\delta }{k_{B}T}-\frac{k_{B}T}{t_{on}R\delta }\exp\left(\frac{F\delta }{k_{B}T}\right)\right),$$

where *t_on_* is the bound lifetime in the absence of force, *R* is the force ramp rate (in our experiments, 2 pN.s^−1^), and *k_B_T* is thermal energy. This equation was fitted to the measured rupture force distribution using the Levenberg–Marquardt nonlinear least squares algorithm and yielded fit parameters of *t_on_* = 1.3 × 10^5^ s (i.e., ~1.5 d) and *δ* = 0.51 nm (see dashed line fit, Fig. 2*G*).

At 20 nM PARP2 concentration, we observe a similar peak at ~85 pN together with a tail of lower values and a small peak at ~45 pN. Efforts to measure the rupture force statistics at lower concentrations were hampered by the low probability of observing a rupture event. The crystal structure shown in Fig. 2*A* reveals no significant interactions between the two PARP2 proteins and so the rupture force for a single PARP2 would be expected to occur at roughly half the value for the dual PARP2 configuration. We therefore interpret the main peak at ~85 pN as due to the formation of the 2:2 stoichiometry of PARP2:blunt ends and the peak at ~45 pN as due to the formation of a single PARP2 bridging from the 5′ phosphate at one end to the opposing 3′ hydroxyl. To further investigate the stoichiometry of PARP2 binding, we investigated the rupture force histogram for a single 5′ phosphate (Fig. 2*G*). Interestingly, we observed a bimodal distribution with two peaks at ~10 pN and ~40 pN. We ascribe the ~40 pN peak to a single PARP2 bridging from a 5′ phosphate to 3′ hydroxyl as observed at 20 nM for 2x5′P. The peak at ~10 pN indicates that there is a second bridging conformation. This conformation has approximately equal likelihood as the 5′ phosphate to 3′ hydroxyl peak at ~40pN given the observed histogram distribution. Since the 0 × 5′P ends showed a complete absence of bridging, we hypothesize that the lower stability state may be due to binding of PARP2 to the single 5′ phosphate but then bridging across to the 5′ hydroxyl rather than the 3′ hydroxyl on the opposing blunt end.

### PARP2 Bridging Restores DNA Torsional Continuity Enabling Supercoiling.

**Fig. 3.**
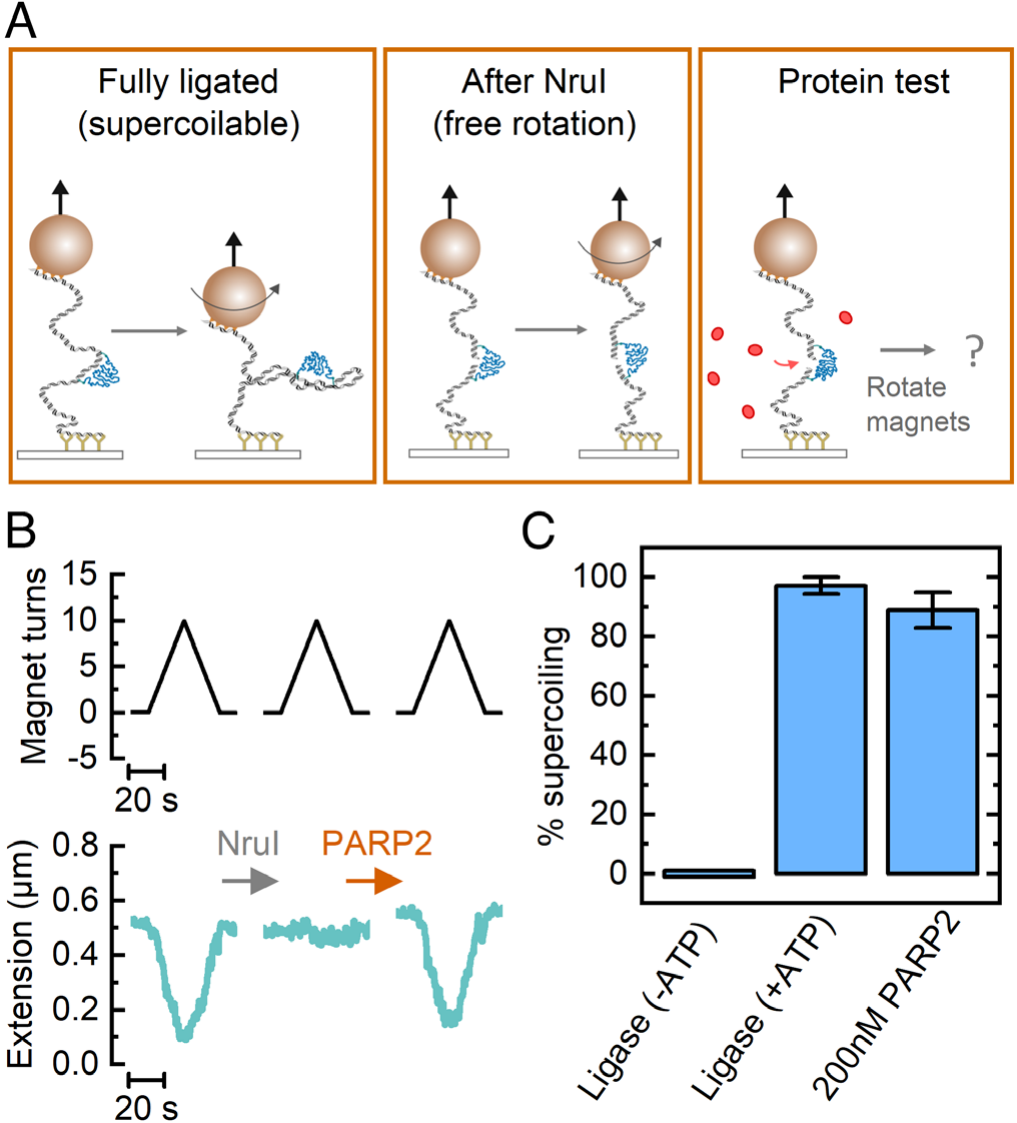
PARP2 binding at a double-strand break restores torsional continuity. (*A*) Schematic of supercoiling test. The force is held constant at 0.4 pN throughout. (*Left*) Initially, the DNA construct is fully ligated and the extension reduces due to plectoneme formation upon magnet rotation. (*Middle*) After formation of a double-stranded DNA break by NruI, the DNA loses torsional continuity and is free to rotate. (*Right*) The protein for measurement is then added to the flow-cell and the magnets are rotated to test whether supercoiling is restored. (*B*) Example experimental trace following the schematics shown in *A*. In this example, PARP2 was added to the flow-cell which resulted in the restoration of DNA supercoiling, indicating that a protein bridge had formed across the broken DNA ends. (*C*) Statistics on percentage of beads in field of view, with supercoiling restored for different test proteins. Error bars show SEM. N = 4 for T4 DNA ligase (−ATP), N = 5 for T4 DNA ligase (+ATP) and N = 3 for 200 nM PARP2.

Having characterized the force-induced rupture of PARP2 bridging across DNA, we next studied the influence of bridging on DNA supercoiling. Magnetic tweezers enables measurement of DNA supercoiling by rotating the magnets, which applies a torque due to preferential alignment of the superparamagnetic bead along its easy axis (34, 35). Fig. 3*A* shows a schematic and example traces of the experimental design we made to test whether PARP2 bridging restores DNA supercoiling. Initially, we measured the fully ligated construct which is torsionally constrained and therefore shows a characteristic decrease in extension when the magnets are rotated and plectonemes form (Fig. 3*B*). After addition of NruI to the flow-cell, we wait until the NruI has created a break at its recognition sequence such that there is no longer a change in extension upon magnet rotation. At the force of 0.4 pN, used throughout, there is no measurable difference in extension after NruI cleavage since this low force is not sufficient to extend the PEG to a measurable degree (*SI Appendix*, Fig. S5). Finally, we add in a test protein, incubate for 1 min, and repeat the magnet rotation. Fig. 3*B* shows an example where we have added PARP2 into the flow-cell and observe that supercoiling is restored, indicating that PARP2 has formed a bridge across the DNA break.

To initially test the system, we performed positive and negative controls using T4 DNA ligase. A concentration of 10 U/μL was used (a typical concentration for bulk DNA ligations in plasmid assembly) and incubated for 1 min in the flow-cell before testing the percentage of beads in the field of view, which show a restoration of supercoiling. Ligase without ATP showed no restoration of supercoiling as expected (Fig. 3*C*). For ligase with 1 mM ATP, we measured that 97 ± 3% (mean ± SEM, N = 5) of beads in the field of view showed a restoration in supercoiling. This control confirms that, at the low force of 0.4 pN used, the broken DNA ends are held in close proximity by the PEG linker, enabling rapid religation. We then repeated the experiment testing the addition of 200 nM PARP2. We observed that 89 ± 6% (mean ± SEM, N = 3) of beads in the field of view showed a restoration of DNA supercoiling when PARP2 was added, thereby showing that PARP2 bridging supports the formation of torsionally constrained DNA molecules.

### Influence of 5′ and 3′ Overhangs on PARP2 Binding.

**Fig. 4.**
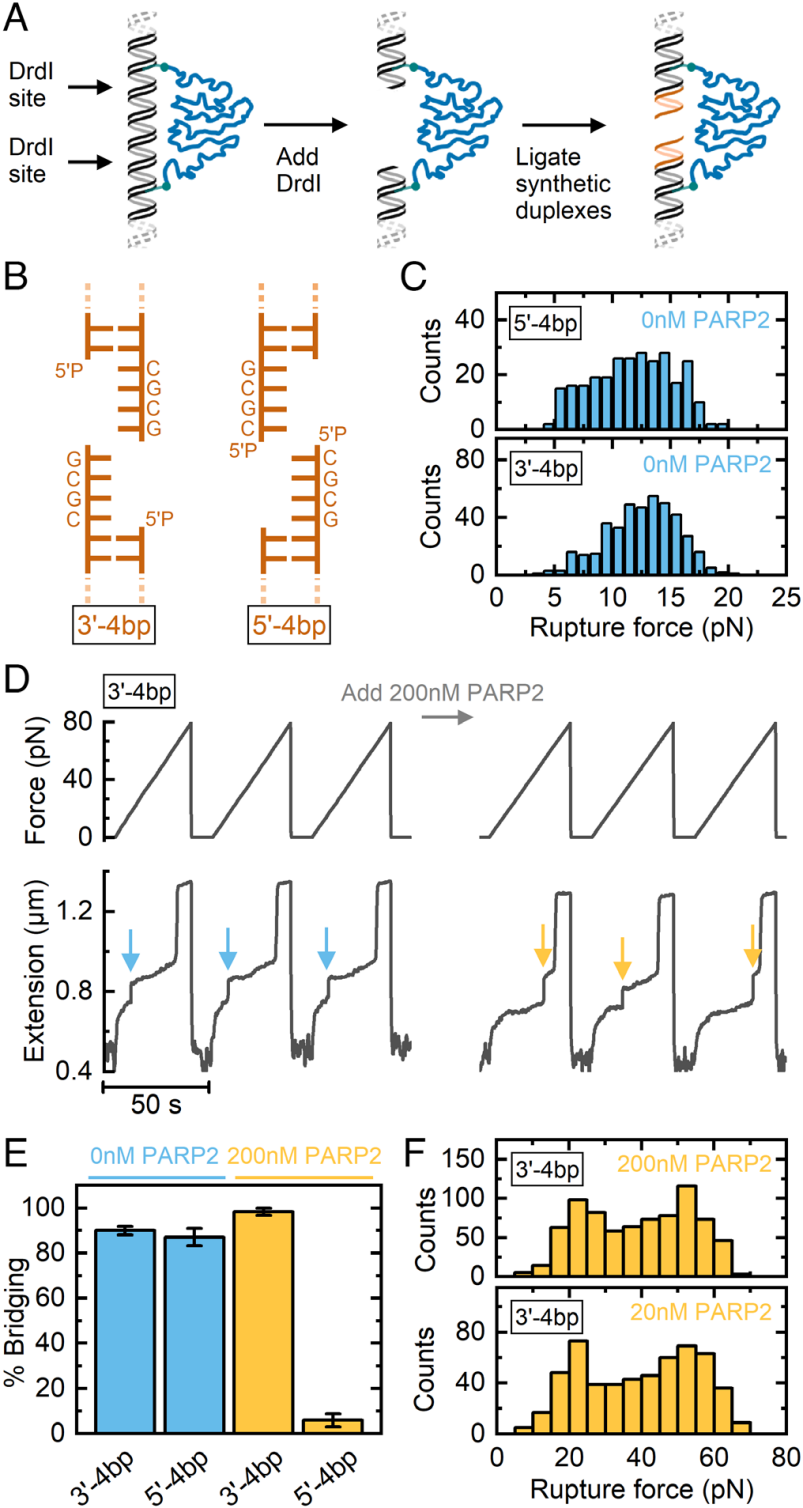
PARP2 switches between binding and bridging modes depending on overhang type. (*A*) Different types of DNA ends are generated by restriction digestion of the initial DNA construct with DrdI followed by ligation of synthetic duplexes. (*B*) Two different types of overhangs assayed—four base overhangs in either the 3′ or 5′ direction as labeled. (*C*) Rupture force histograms for both overhang types in the absence of PARP2. Data are cumulative from N = 3 beads (5′-4 bp) and N = 4 beads (3′-4 bp). (*D*) Example data trace showing force ramps before and after adding 200 nM PARP2 using the 3′-4 bp design. The arrows mark the points of rupture. (*E*) Percentage bridging observed in force–extension curves for 3′ and 5′ overhangs for 0 nM and 200 nM PARP2 (in order from *Left* to *Right*: N = 4, N = 3, N = 4, and N = 9). Error bars show SEM. (*F*) Rupture force histogram for 3′-4 bp overhang in the presence of 20 nM PARP2 (cumulative data from N = 3 beads) and 200 nM PARP2 (cumulative data from N = 4 beads).

Having tested the binding of PARP2 to blunt DNA double-strand ends, we investigated the influence of different overhang structures. Our DNA construct features two restriction sites for the enzyme DrdI at equidistant positions from the PEG linkages (Fig. 4*A*). Similar to NruI (described above), cleavage of the DNA could be observed in real time as a sudden increase in DNA extension after addition of DrdI to the flow-cell (*SI Appendix*, Fig. S9). After cleavage, we used the overhangs formed by DrdI to ligate on different synthetic duplex structures in situ in the magnetic tweezers’ flow-cell. Specifically, we made structures with 5′ and 3′ overhangs of four base lengths and with the same 5′-CGCG-3′ sequence (Fig. 4*B*). We then used our magnetic tweezers assay to perform force ramps and measure the rupture force as described in Fig. 2. Fig. 4*C* shows histograms of the rupture force for both 3′ and 5′ overhangs (in the absence of PARP2). Both overhang types produce readily detectable rupture events, due to complementary base pairing, with forces between 5 and 20 pN. Fig. 4*D* shows an example trace comparing force ramps before and after adding 200 nM PARP2 to the 3′-4 bp design. The arrows mark the increase in extension corresponding to when the bridging of the ends ruptures and the PEG linker is extended.

In Fig. 4*E*, we compare the percentage of force ramp curves showing a rupture event for both 3′ and 5′ overhangs (at 0 nM and 200 nM PARP2). For the 3′ and 5′ overhangs with 0 nM PARP2, we observe that 90 ± 2% and 87 ± 4% (mean ± SEM, N = 4 and N = 3 beads, respectively) of force ramps show a rupture event (due to base-pair hybridization). Similarly, after addition of 200 nM PARP2 to the 3′ overhang structure, we observed a high percentage of bridging of 98 ± 2% (mean ± SEM, N = 4) and with a significantly enhanced rupture force (Fig. 4 *D* and *F*). However, after addition of PARP2 to the 5′ overhang DNA construct, rather than an enhancement in bridging rupture force, we observed that bridging was almost completely blocked at 6 ± 3%, (mean ± SEM, N = 9). This indicates that PARP2 no longer bridges across the broken DNA ends but instead binds in a manner that blocks complementary base-pairing between the two ends (see *SI Appendix*, Fig. S10 for example force ramp trace).

In Fig. 4*F*, we show the rupture force histograms after addition of 20 nM or 200 nM PARP2 to the four base 3′ overhang constructs. We observe a clear increase in rupture forces compared to 0 nM PARP2 (Fig. 4*C*), indicating the formation of a bridging interaction by PARP2. Using overhangs with 5′ hydroxyl groups rather than 5′ phosphate completely negated this effect, again showing that PARP2 bridging is specific to the presence of 5′ phosphate (*SI Appendix*, Fig. S11). The rupture force histograms in Fig. 4*F* show bimodal distributions, and the shape of the distribution does not change significantly when increasing the concentration from 20 nM to 200 nM. This indicates that the two peaks correspond to distinct states of bound PARP2 (rather than having one or two bound PARP2 as was observed for 2 × 5′P blunt ends in Fig. 2*G*). Taking the crystal structure of PDB:7aeo (shown in Fig. 2*A*) and simulating an overhang in the 3′ direction indicates significant steric clashes if two PARP2 proteins are bound across each nick on the DNA (*SI Appendix*, Fig. S12). This suggests that it is unlikely that two PARP2 proteins can bind to hybridized DNA overhangs—potentially explaining why the rupture forces are lower than those observed for blunt-ended DNA. However, the exact conformational details of the two states remain to be determined.

### PARP1 Does Not Bridge DSBs and Competes away PARP2 in an Open DSB Conformation.

**Fig. 5.**
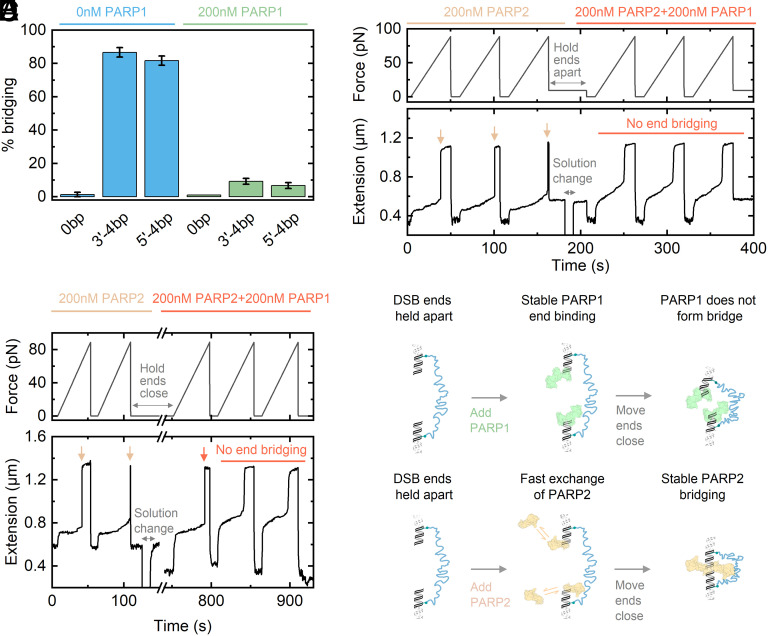
PARP1 does not bridge double-strand breaks and competes away PARP2 at open DSB ends. (*A*) Percentage bridging measured at 0 nM PARP1 and after addition of 200 nM PARP1 for different end types. N = 5 for 0 bp and N = 8 for both 3′-4 bp and 5′-4 bp. Error bars show SEM. (*B*) Trace comparing force ramp responses for 200 nM PARP2 before subsequent addition of 200 nM PARP2 + 200 nM PARP1 for blunt-end 5′ phosphorylated ends. The DSB ends are held apart using a force of 9 pN during solution change. The arrows indicate PARP2 bridge ruptures—after solution change, bridging events are no longer observed. (*C*) In a similar experiment to *B*, the solution was exchanged to contain 200 nM PARP2 + 200 nM PARP1, but the ends were held in close proximity. After incubation for 10 min, force ramps were used to test for bridging. Force rupture of a PARP2 bridge was observed in the first ramp but not in subsequent ramps. (*D* and *E*) Models for PARP1 and PARP2 interactions at blunt-end 5′ phosphorylated DNA ends. PARP1 stably binds at such DSB ends and does not form a bridge when the ends are brought in close proximity. In contrast, PARP2 rapidly exchanges at open DSB ends but forms a highly stable bridging structure when the ends come together.

After characterizing the binding and bridging of PARP2 across different types of DNA DSBs, we investigated the behavior of PARP1. We repeated the tests described in Figs. 2*F* and 4*E* where we perform multiple force ramps after addition of protein and then determined the percentage of these force ramps which show bridging of the two ends. Fig. 5*A* shows the result of these experiments for three types of overhangs—blunt ends (0 bp), 3′-4 bp, and 5′-4 bp (both phosphorylated and with same sequences as Fig. 4). For the blunt ends, we did not observe PARP1 bridging across the two ends in the presence of 200 nM PARP1. Furthermore, PARP1 blocked bridging due to the presence of complementary base-pair overhangs for both 3′-4 bp and 5′-4 bp—with <10% of force ramps showing a rupture event—similar to the activity of PARP2 on the 5′-4 bp overhang (Fig. 4*E*).

We also performed competition experiments between PARP2 and PARP1. Fig. 5*B* shows an example trace where we compare behavior under force with 200 nM PARP2 only before adding 200 nM PARP1 + 200 nM PARP2. With 200 nM PARP2 only, we observe the characteristic high force rupture events of PARP2 bridging as indicated by the arrows (Fig. 2*D*). We then held the magnetic bead at 9pN while adding solution such that the ends are held apart during this time, preventing the formation of a PARP2 bridge. Having added the new solution of 200 nM PARP1 + 200 nM PARP2, we perform additional force ramps. These force ramps show an absence of end-bridging—the only sudden increase in extension during the ramp is due to the 65 pN B–S transition. This indicates that PARP1 rapidly competes away PARP2 when the ends are held in this open conformation. We similarly observed this effect when using calf-intestinal phosphatase rather than PARP1 as a competitor (*SI Appendix*, Fig. S13)—where the lack of bridging now signifies that phosphatase can rapidly access the 5′ phosphate which is crucial for PARP2 bridging.

In a second approach, we repeated the competition assay with PARP1 but this time held the ends in close proximity before solution exchange such that the PARP2 bridge could reform (Fig. 5*C*). Here, we observed that the first force ramp after PARP1 addition showed the characteristic high force rupture of a PARP2 bridge before subsequent ramps had an absence of end-bridging. This was measured even after incubating PARP1 for 10 min before applying subsequent ramps [an extension decrease is observed during the 10 min incubation due to DNA condensation by PARP1 (36)]. Overall, this experiment indicates that when a PARP2 bridge has already formed, it is not readily displaced by PARP1.

In Fig. 5 *D* and *E*, we present models for PARP1 and PARP2 at blunt 5′ phosphate DSB ends. If the ends are apart (e.g., being held apart under force), then PARP2 exchanges rapidly at the DSB as evidenced by its fast displacement by PARP1 and the activity of calf-intestinal phosphatase. However, if the two DSB ends are in close proximity, such that the ends are able to interact, PARP2 can form a highly stable bridging interaction which resists mechanical forces up to 90 pN (Fig. 2*G*). PARP1, in contrast, binds stably at DSB ends but does not form a bridging interaction when the two ends are brought together.

## Discussion

In summary, we have used a magnetic tweezers assay to measure the end-binding and bridging of PARP1 and PARP2 at DNA DSBs. Our assay allows us to perform highly controlled experiments using single-molecule manipulation to control the distance between two DSB ends and to measure the rupture force of proteins that bridge across the break. Our results confirm recent structural work revealing that PARP2 can form a bridged structure across 5′ phosphorylated blunt DNA ends (37). Moreover, we have shown that this bridge restores torsional continuity to DNA and forms a remarkably stable interaction with a rupture force distribution in the range of 60 to 95 pN for the dimeric form. This rupture force is significantly greater than the 20 to 30 pN required to irreversibly unwrap a nucleosome from DNA (38–40), the ~25 pN stall force of RNA polymerase (41), or even the stall force of the strongest known DNA molecular motors such as the ϕ29 packaging motor at ~60 pN (42). The high mechanical strength of this interaction indicates that it could cause the formation of stable roadblocks which would impede normal genomic processes at a double-strand DNA break.

In addition to measuring blunt-ended DNA, we tested the effect of dephosphorylating the 5′ end as well as DNA double-strand breakage that results in 5′ or 3′ overhangs. Our experiments show that PARP2 bridging is highly specific to 5′ phosphorylated DNA. Interestingly, we measured a switch between binding and bridging for the same DNA sequence overhang depending on whether it was in the 5′ or 3′ direction. We also observed multiple peaks in rupture force histograms in the presence of PARP2 in the context of different overhang types. These measurements show a rich variety of interaction modes depending on the exact configuration of the DNA break. The observation that short 3′ overhangs also show stable PARP2 bridging indicates that it might be an important feature during end-resection processes.

Our experiments also revealed that PARP1 did not form a bridge linking together the two ends and it competed away PARP2 bridge formation. This observation reveals a notable difference in activity between the two proteins at DSBs. Currently, clinically approved PARP inhibitors target both PARP1 and PARP2—with more specific PARP1 inhibitors under development (43). Literature reports have also shown that PARP1 and PARP2 differ in that PARP2 specifically requires 5′phosphate for poly(ADP-ribose) synthesis (7). Our results show further differences in the basic mechanisms of DNA interactions between PARP1 and PARP2 that might be exploited in PARP inhibitor therapy.

Finally, the PEG–DNA synthesis and force-spectroscopy platform we have established has potential for the study of other proteins involved in DNA DSB repair. In the context of PARP biochemistry, our approach could be extended to test for small-molecule inhibitor effects and examine how other proteins compete for binding. More broadly, the technique could be used to study a wide range of protein complexes that are known to bind across DNA ends such as those involved in nonhomologous end-joining and microhomology-mediated end joining.

## Methods

### Protein Expression and PEG–DNA Synthesis.

Human PARP1 and PARP2 proteins were made using a baculovirus expression system and purified by Immobilized Metal Affinity Chromatography (IMAC) affinity and size-exclusion chromatography (see *SI Appendix*, Fig. S14 for further details). The PEG–DNA construct synthesis is described in detail in *SI Appendix*, Figs. S1–S3. Briefly, a 20 kDa bis-azide PEG was reacted overnight with a Dibenzocyclooctyne (DBCO) modified oligonucleotide before agarose gel purification. The full-length DNA construct was then generated in a golden-gate assembly reaction (44) with PCR amplicons before a final agarose gel purification.

### Flow-Cell Construction.

Flow-cell fabrication is detailed in depth in a previous publication (45). Briefly, the flow-cell is assembled using silicone tape, glass coverslips, and polydimethylsiloxane. Solutions are flowed through using a gravity-feed method and flow is automatically arrested by a capillary-action stop valve. The bottom coverslip is coated sequentially with nitrocellulose and antidigoxigenin (11333089001, Sigma Aldrich) before cross-linking with glutaraldehyde and passivating with BSA (A0281, Sigma-Aldrich) and β-casein (C6905, Sigma-Aldrich). Three micrometers-sized latex beads (LB30, Sigma-Aldrich) are fixed to the surface, which act as fiducial markers for drift correction.

### Magnetic Tweezers Measurements.

Superparamagnetic beads (M-280 streptavidin Dynabeads, ThermoFisher) were incubated with gel-purified DNA constructs before introducing into the flow-cell. The beads were imaged by collimated LED illumination (ThorLabs, M625L4-C4) with a 40× or 60× Nikon oil-immersion objective and a monochrome camera (Imaging Source, DMK 33GX249). We used a pair of 5 mm × 5 mm × 5 mm N50 cube magnets (C0057, SuperMagnetMan) in one of the following two configurations: 1) with a 2-mm gap, mounted on a rotating motor to enable DNA supercoiling, and 2) with a 0.3-mm gap. The magnets used for each experiment and calibration methods are described in *SI Appendix*. The magnetic beads were tracked in real time at 20 frames per second using a previously described video image analysis algorithm (46) with custom C++ and LabVIEW (National Instruments) software. Traces displayed in figures were filtered with a five-point median filter. All measurements with PARP proteins were in a buffer of 20 mM Tris-HCl (pH 7.5), 150 mM NaCl, 2 mM MgCl_2_, 0.5 mM Tris (2-carboxyethyl) phosphine (TCEP), 1 mg/mL Bovine Serum Albumin (BSA), and 1 mg/mL β-casein. NruI and DrdI cleavage was performed in 1× CutSmart buffer New England Biolabs (NEB) supplemented with 1 mg/mL BSA, 1 mg/mL β-casein. Tests of T4 DNA ligase (Fig. 3) were performed in 1× NEB CutSmart (−ATP buffer) or 1× NEB T4 ligase buffer (+ATP buffer) with a concentration of 10 U/μL. All experiments were conducted at room temperature (22 ± 1) °C.

## Supplementary Material

Appendix 01 (PDF)

## Data Availability

All data associated with the manuscript are provided at crick.figshare.com data have been deposited in crick.figshare.com (10.25418/crick.22644118) (47).
