# Supplementary material for: Single-molecule force spectroscopy reveals binding and bridging dynamics of PARP1 and PARP2 at DNA double-strand breaks
Source: Proc Natl Acad Sci U S A. 2023 May 22;120(22):e2214209120. doi: 10.1073/pnas.2214209120 (PMC10235982; doi:10.1073/pnas.2214209120)
Supplement: Supplementary file 1 — Appendix 01 (PDF) [file pnas.2214209120.sapp.pdf]

Supporting information

Single-molecule force spectroscopy reveals binding and bridging dynamics of PARP1 and PARP2 at DNA double-strand breaks

Nicholas A. W. Bell[1,2*] and Justin E. Molloy[1,3]

[1]The Francis Crick Institute, 1 Midland Road, London, NW1 1AT, UK

[2]University College London, Gower Street, London, WC1E 6BT, UK

[3]Centre for Mechanochemical Cell Biology, Warwick Medical School, Coventry, CV4 7AL

* To whom correspondence should be addressed.

Email: n.a.w.bell@ucl.ac.uk

This PDF file includes:

Fig. S1 - Synthesis of DNA with central PEG linker
Fig. S2 - Gel analysis of copper-free click coupling of DNA and PEG
Fig. S3 - Gel analysis after golden gate reaction
Fig. S4 - Molecular weight dependence of PEG mean end-to-end distance
Fig. S5 - Worm-like chain model of PEG
Fig. S6 - Magnetic tweezers trace showing action of NruI on multiple beads in the field of view
Fig. S7 - Antarctic phosphatase treatment for removal of 5' phosphates
Fig. S8 - Limiting kinase treatment to generate single 5' phosphate
Fig. S9 - Magnetic tweezers trace showing action of DrdI on multiple beads in the field of view
Fig. S10 - Absence of end bridging for 5'-4bp overhang in presence of 200 nM PARP2
Fig. S11 - PARP2 does not bind at overhang sequences with 5'OH
Fig. S12 - Extrapolation of PDB:7aeo to 5' and 3' overhangs
Fig. S13 - PARP2 bridging is removed when adding phosphatase and holding ends apart
Fig. S14 - SDS-PAGE gel of PARP1 and PARP2

Supplementary Text Section - Calibration of magnetic tweezers

Table S1 - Primers for PCR amplification of DNA spacers and handles
Table S2 – Duplex sequences for generating 5' and 3' overhangs

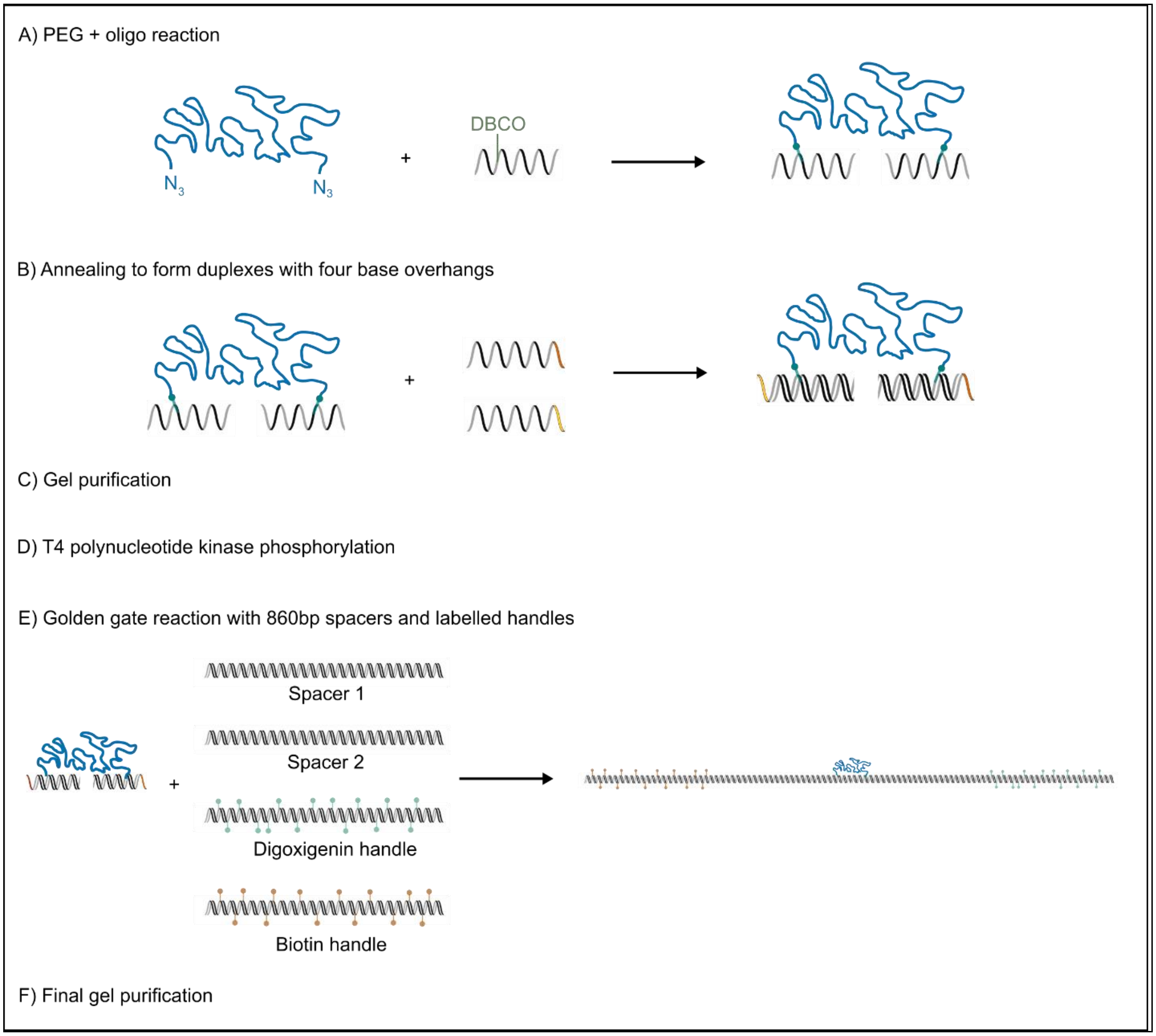

Figure S1. Synthesis of DNA with central PEG linker. The figure shows the steps used to synthesise the DNA linker with a central bridging 20 kDa PEG. (A) An oligonucleotide with an internal DBCO modification at a thymine base was purchased from Biomers. The sequence was:

*5′-CGATTTGACCTAGTCGTCAAACG**X**TCTGCTAG-3′*

Where 'X' indicates a DBCO modified thymine. This oligo was reacted overnight with a 20 kDa azide terminated homobifunctional PEG (756601-1G, Sigma-Aldrich) at 37ºC. (B) An equimolar mixture of oligos with sequence *5'-PhosCAATCTAGCAGAACGTTTGACGACTAGGTCAAATCG-3'* and *5'-PhosCCCACTAGCAGAACGTTTGACGACTAGGTCAAATCG-3'* was then added and annealed by heating to 70ºC before cooling to room temperature over 15 minutes. The red letters indicate the four base overhangs formed. (C) The sample was then run on a gel and the band corresponding to two linked duplexes was purified (see Figure S2). (D) The purified, linked duplexes were then phosphorylated using T4 polynucleotide kinase. (E) 860 bp long spacers and handles were prepared by PCR amplification of a section of lambda DNA. The handles contained either biotin or digoxigenin groups by including labelled

dUTP nucleotides in the PCR at a ratio of 1:8 of dUTP:dTTP. The primers used in the PCR amplification are given in Table S1. Primer overhangs were designed with a recognition site for BsaI and four base overhangs designed to form after BsaI digestion. The ligation of the spacers, handles and linked duplexes was carried out in a single step "golden gate" reaction (1) using the simultaneous action of BsaI and T4 DNA ligase. (F) The assembled construct was then gel purified (Figure S3).

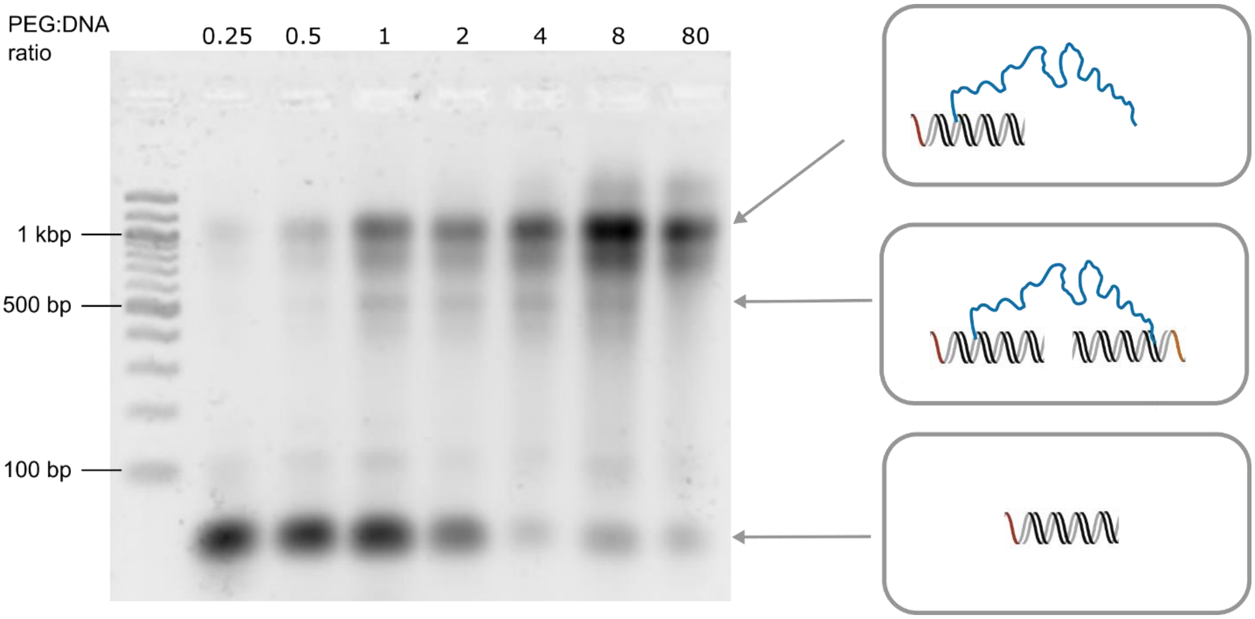

Figure S2. Gel analysis of copper-free click coupling of DNA and PEG. 2% agarose gel showing the product formed after reacting the DBCO and bisazide PEG and annealing to form a duplex ie after stage B in Figure S1. The PEG was incubated with the DBCO labelled DNA oligo in increasing ratios as marked above each lane. Three bands identified as single duplex only, single duplex with one PEG attached and two duplexes linked by one PEG are shown. The band assignment for the two duplexes linked by one PEG was based on the observation that this band disappeared at a high ratio of excess PEG (80x). Somewhat surprisingly, this band runs faster than the band for a single duplex – indicating that the PEG linker has a significant effect on electrophoretic mobility. This figure shows an analysis gel stained with gelred for imaging with UV light. For the full synthesis method, a 2:1 or 1:1 ratio of PEG to DNA was used and the band corresponding to the two linked duplexes was imaged and extracted using staining with gelgreen (Biotium) and blue light illumination to avoid UV light induced damage.

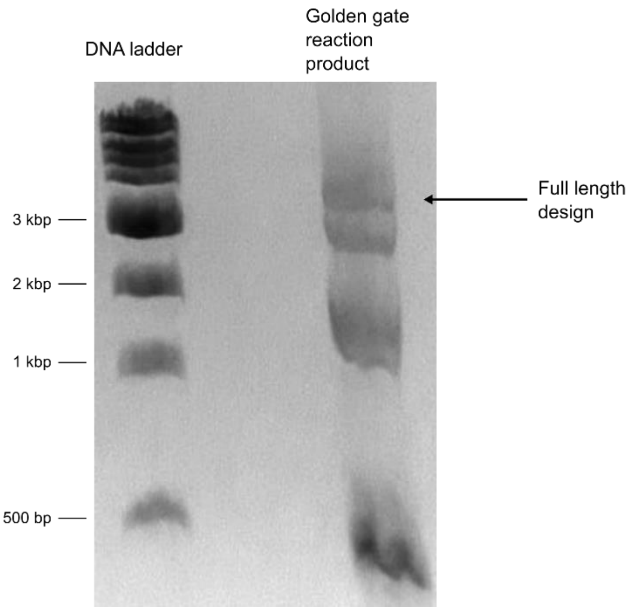

Figure S3. Gel analysis after golden gate reaction. The product after stage E in the synthesis scheme (Figure S1) was run on a 1% agarose gel. The gel was visualized with gelgreen and blue light illumination and the top band was extracted for magnetic tweezers experiments.

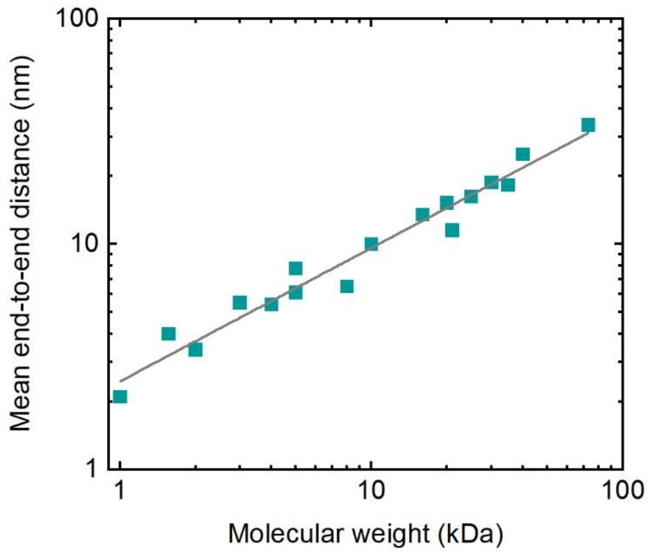

Figure S4. Molecular weight dependence of PEG mean end-to-end distance. Data points are from the appendix of the paper by Sherck *et al* (2) who determined a power law fit to the data $M_{e\text{-}e} = 0.047(M_w)^{0.588}$.

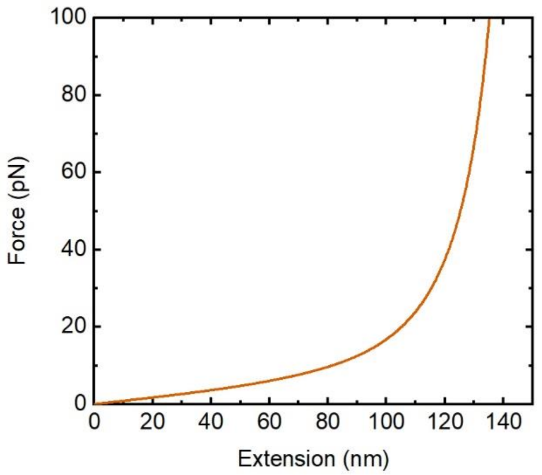

Figure S5. Worm-like chain model of PEG. The curve shows the seventh-order expansion approximation for the worm-like chain model given in (3). The value used for the persistence length of PEG was 0.45 nm (2) and the monomer length was 0.35 nm (4). 20 kDa PEG has 454 monomer units and the contour length is therefore 159 nm. The extension at 8 pN is 72 nm according to this model. The attachment points of the PEG on the DNA are separated by 16 nm and therefore the expected extension change after cutting with NruI is 56 nm.

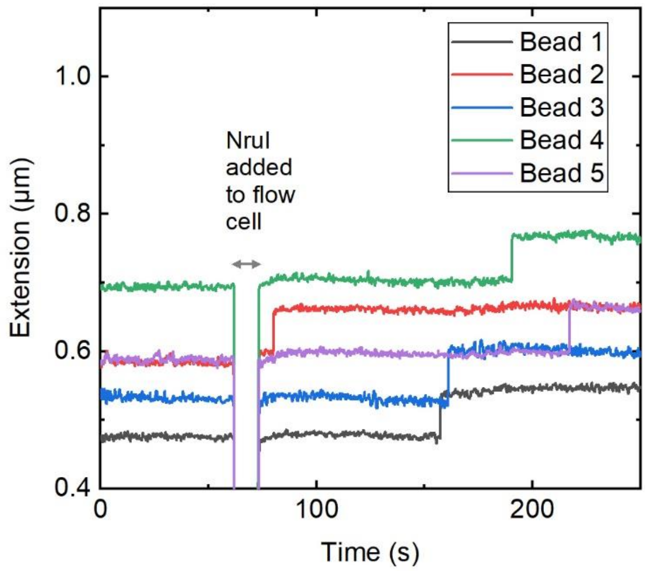

Figure S6. Magnetic tweezers trace showing action of NruI on multiple beads in the field of view. The trace shows the same data as in Figure 1C in the main text but with multiple bead traces (Only Bead 1 is shown in the main text). In each case, a step change in extension is observed after adding NruI indicating the successfully assembly and digestion of the PEG linked DNA construct. The bead-to-bead differences in offset are due to different off-axis attachment points on the tethers (5).

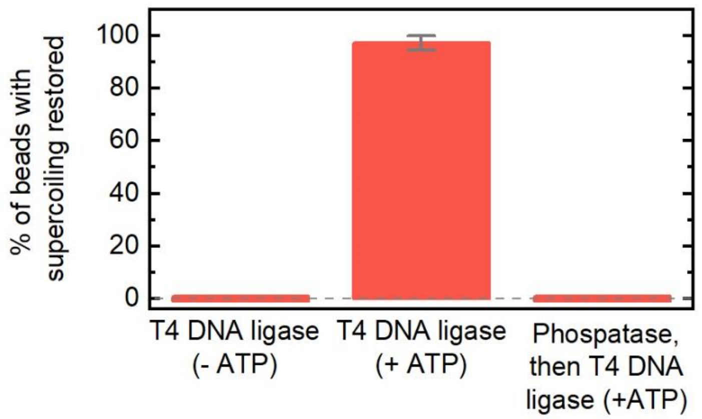

Figure S7. Antarctic phosphatase treatment for removal of 5' phosphates. After cleavage by NruI, the DNA has blunt end 5'-phosphorylated ends. To remove the 5' phosphates (thereby generating 5' hydroxyls), we added antarctic phosphatase (M0289S, New England Biolabs) at a concentration of 1.25U/µL (in 1x antarctic phosphatase buffer, B0289S, New England Biolabs) and incubated for 2 minutes. To check that this successfully resulted in the removal of 5' phosphates, we ran tests where we then added T4 DNA ligase at 10 U/µL and examined whether supercoiling was restored (ie the same assay as shown in Figure 3 in the main text). The first two columns are as shown in Figure 3 in the main text – comparing ligase with and without ATP. In three independent experiments where we add antarctic phosphatase before addition of ligase, we observed no restoration of supercoiling after the ligase step (N=21 total beads measured) indicating that the phosphatase has removed the 5' phosphates preventing religation. This same incubation method was used for the 0x5'P measurements shown in Figure 2 in the main text.

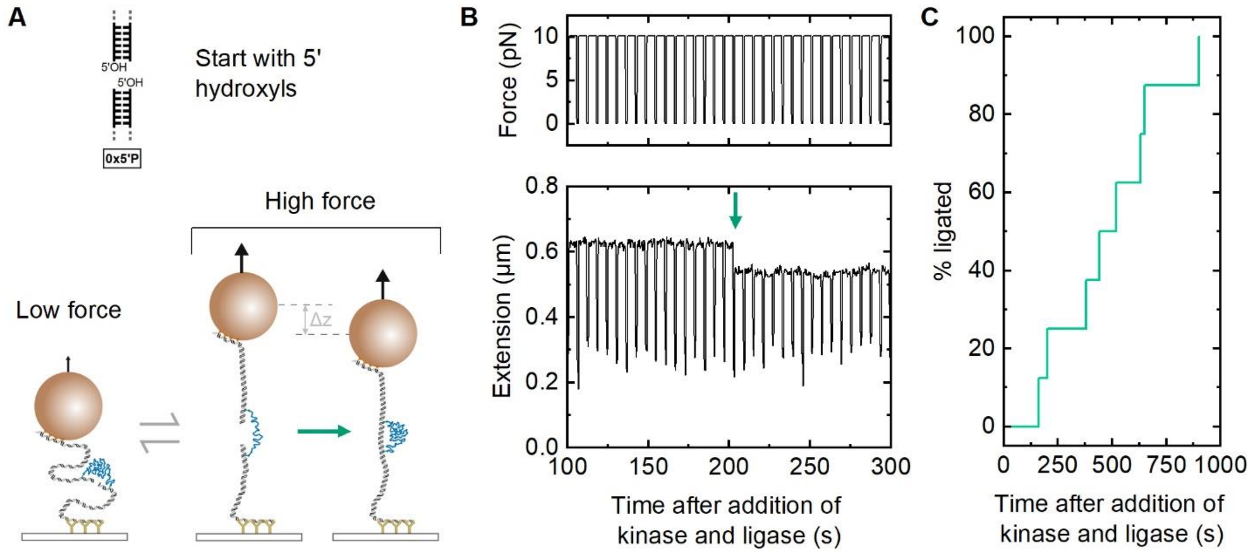

Figure S8. Limiting kinase treatment to generate single 5' phosphate. To generate single 5' phosphate ends, we developed a protocol where, having removed the phosphates with antarctic phosphatase, we

incubated with a limited kinase concentration. We used this technique (rather than simply adding a low concentration of antarctic phosphatase after NruI cleavage) since it afforded a simple method for determining the appropriate concentration of kinase and incubation time: after antarctic phosphatase treatment, we added a high concentration of ligase together with a low concentrations of kinase. T4 DNA ligase (M0202S, New England Biolabs) at 10U/µL was used together with T4 polynucleotide kinase (M0201S, New England Biolabs). A 1x T4 buffer was used (B0202S, New England Biolabs) in which both enzymes are active. We then applied a square wave force modulation where we alternated between ~0.05pN and 10pN for 1s and 5s continuously (Figure S8A). Using this technique, we can determine the time required for 5' phosphate addition by observing the step change in DNA extension that results from ligation (which is relatively rapid, due to the high concentration of ligase). Figure S8B shows an example where we added 0.0025 U/µL T4 polynucleotide kinase and measured the ligation on one bead. Figure S8C shows the time measured before ligation for eight beads using 0.0025 U/µL T4 polynucleotide kinase. The median value was 480s. For experiments measuring rupture for a single phosphate (Figure 2) we incubated for the median time (with 0.0025 U/µL kinase only) before a high salt wash to remove the kinase and ligase. DNA molecules with a single phosphate were readily distinguished by the lower characteristic rupture forces (~5-55 pN) compared to dual phosphate (~60-95pN) at 200 nM PARP2.

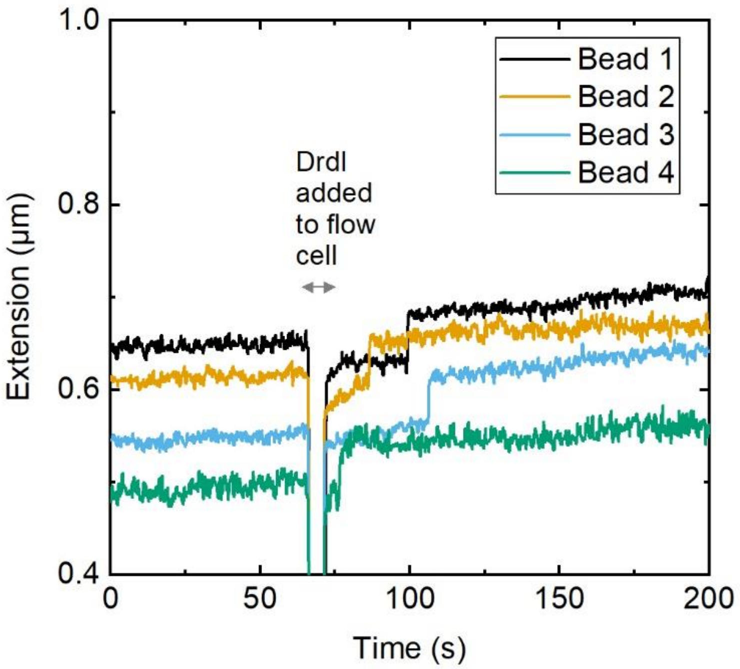

Figure S9. Magnetic tweezers trace showing action of DrdI on multiple beads in the field of view. Example trace showing four beads and action of DrdI in creating a DSB after addition to the flow cell. Each bead shows a step change in extension after DrdI addition indicating successful DrdI cleavage.

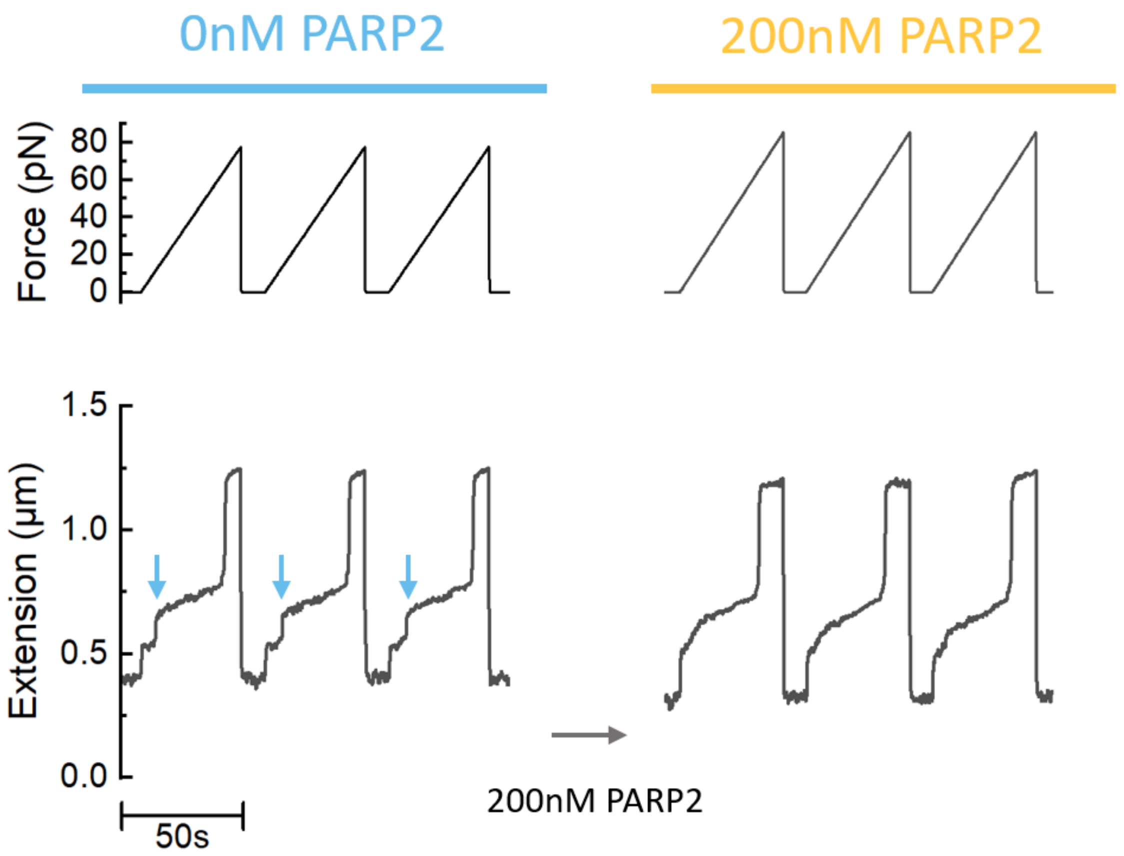

Figure S10. Absence of end bridging for 5'-4bp overhang in presence of 200 nM PARP2. Figure shows an example trace comparing force ramps before and after adding 200 nM PARP2 for the 5'-4bp structure (Figure 4 in main text). Before addition of PARP2 the characteristic rupture of the 4 base overhang is observed. After addition of PARP2, bridging is no longer observed. Note: in this experiment the force ramp was to a slightly higher peak value after addition of 200nM PARP2.

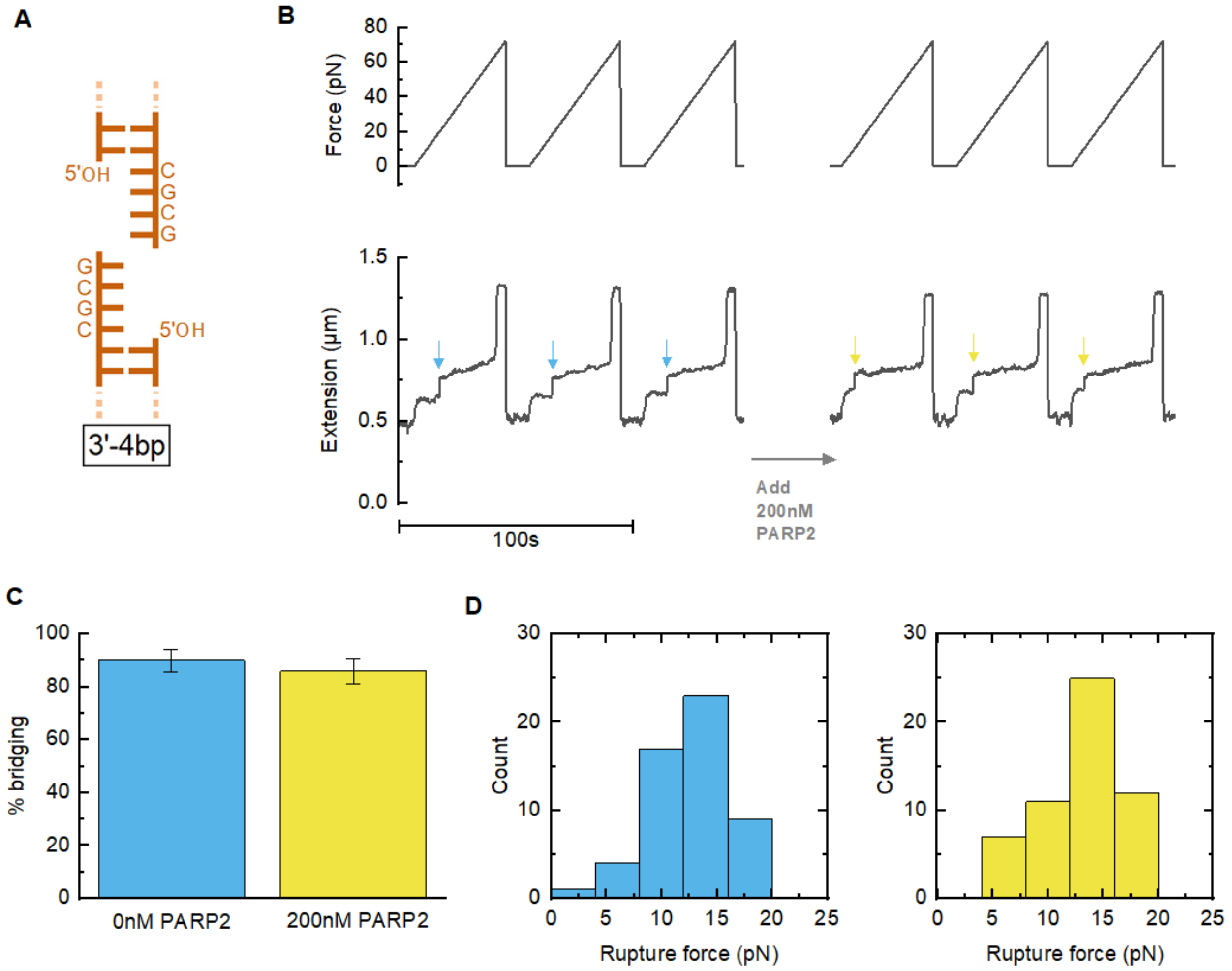

Figure S11. PARP2 does not bind at overhang sequences with 5'OH. (A) The 3'-4bp overhang sequence shown was tested for PARP2 bridging. The sequence is the same as used in Figure 4 but with 5' hydroxyls rather than 5' phosphates. (B) Trace showing force ramps before and after addition of 200 nM PARP2. (C) Comparison of % bridging before and after adding 200 nM PARP2. (D) Force rupture histograms before and after adding PARP2 show no change thereby indicating that PARP2 bridging is specific to the presence of 5' phosphorylation.

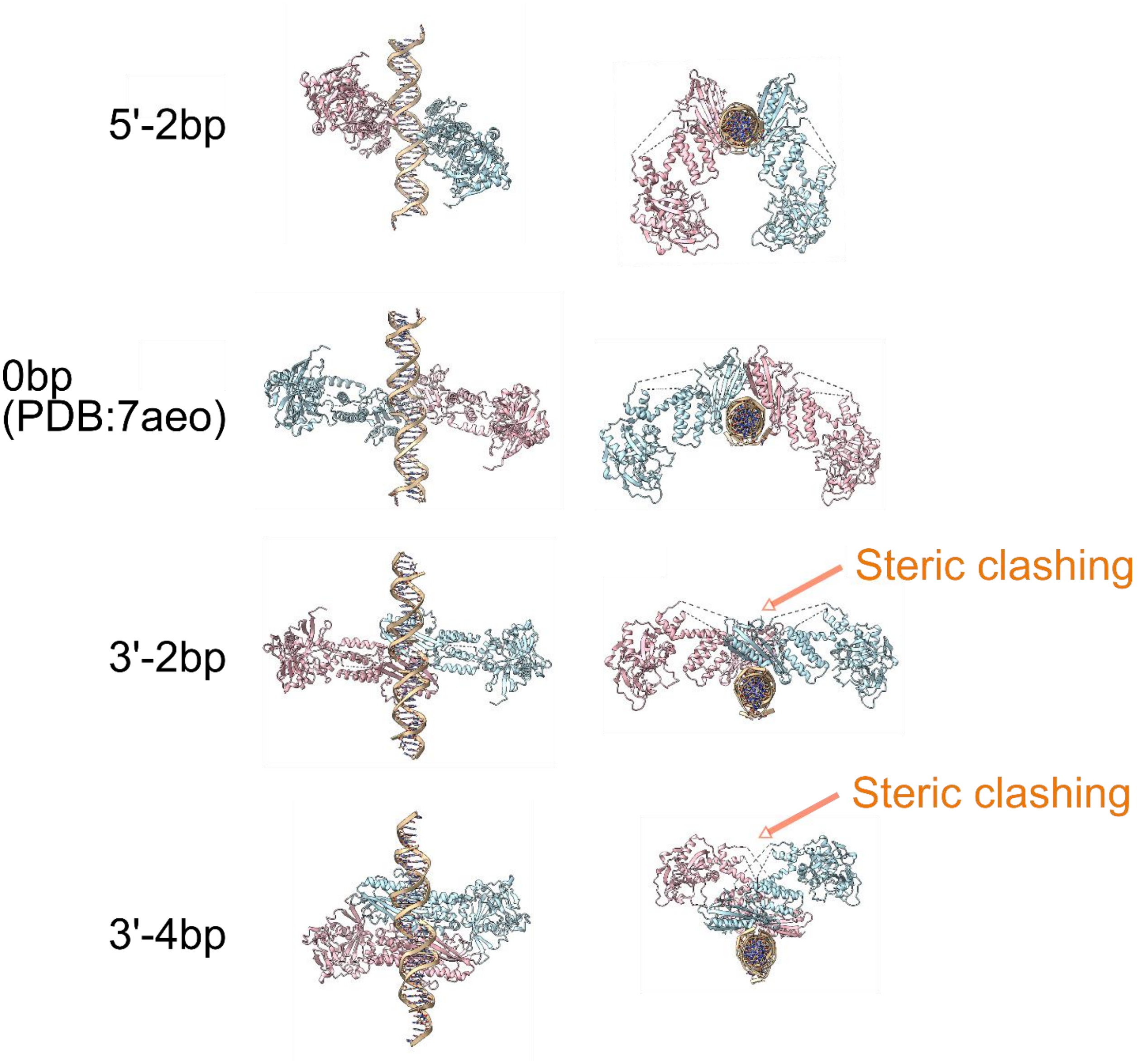

Figure S12. Extrapolation of PDB:7aeo to 5' and 3' overhangs. The effect of overhangs was simulated by translating PARP2 proteins along the helical path of DNA by two basepairs in either 5' or 3' direction. The left and right columns show orthogonal views. An overhang in the 3' direction creates substantial steric clashes between the WGR domains of the two PARP2 proteins.

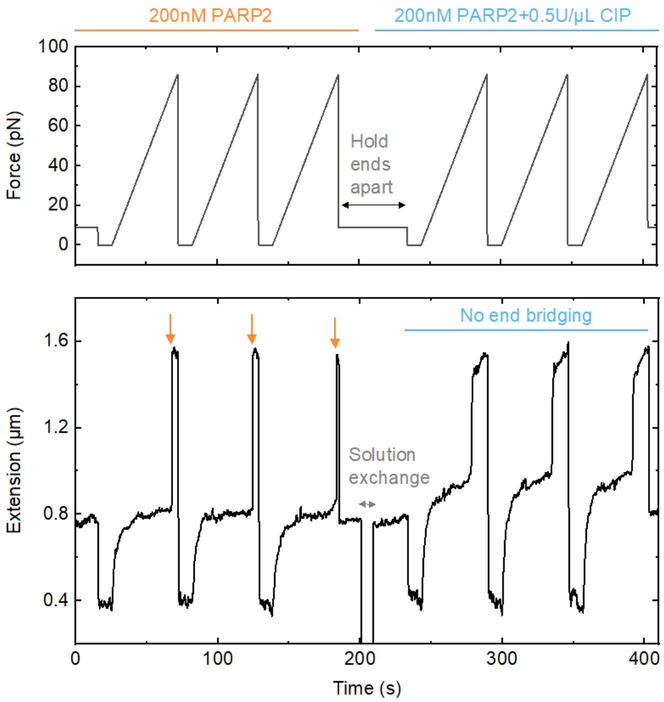

Figure S13. PARP2 bridging is removed when adding phosphatase and holding ends apart. Experiment was performed in a buffer of 1x cutsmart (New England Biolabs) with 1mg/ml BSA and 1mg/ml β-casein. With 200nM PARP2 in solution, we observe characteristic bridging which ruptures under force (as marked by the orange arrows). With the ends held apart by a force of 9 pN, we exchange to a solution of 200nM PARP2 + 0.5 U/μL Quick CIP (NEB). We then observe an absence of bridging interactions with the overstretch transition occurring at 65 pN.

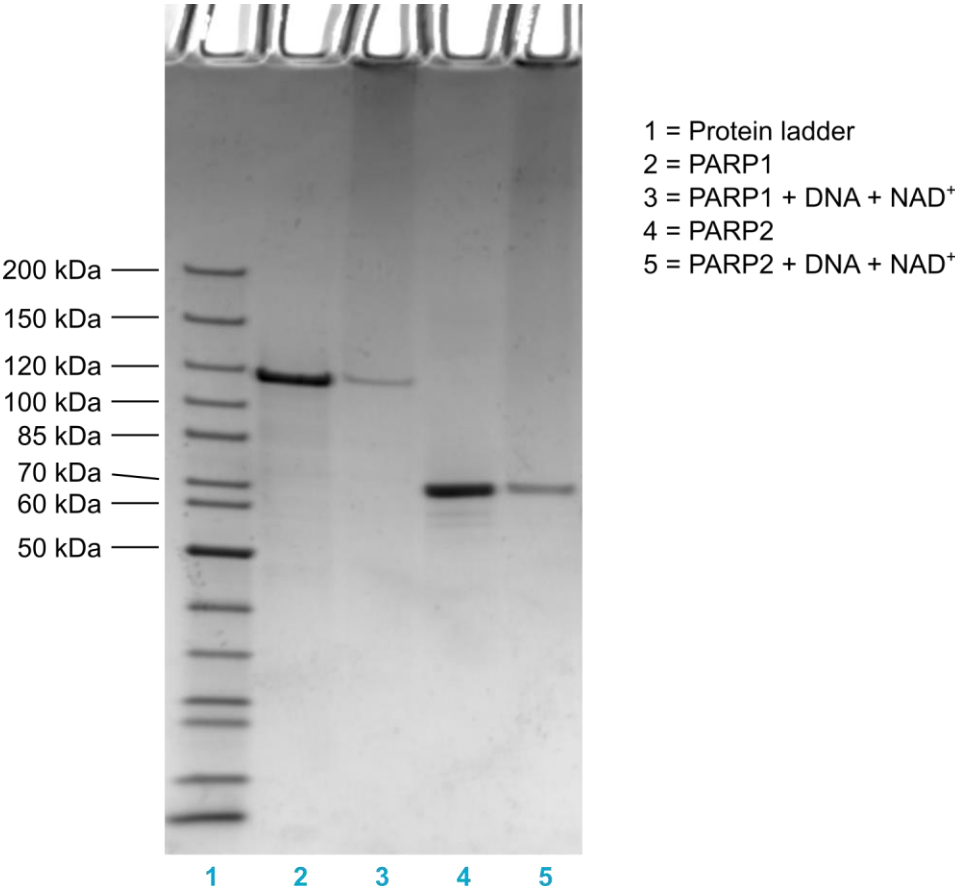

Figure S14. SDS page gel of PARP1 and PARP2. The human PARP1 sequence (accession # AAH37545) was cloned into a pFastBac expression vector to include a N-terminal 6His-6Lys-TEV tag & cleavage site. P1 virus was generated and protein expressed following the standard Invitrogen protocols. Similarly, the human PARP2 sequence (accession #NP_005475) was cloned into a pFastBac expression vector to include a N-terminal Avi-6His-TEV tag & cleavage site. P1 virus was generated and protein expressed following the standard Invitrogen protocols. PARP1 and PARP2 were purified in a two-step process; firstly a IMAC affinity purification and secondly a Size Exclusion Chromotography step on an AKTA system. The final protein was stored in aliquots at -80ºC. The purified PARP1 and PARP2 proteins used in this study were characterized by SDS-PAGE gel analysis. Lanes 2 and 4 show the purified proteins. Lanes 3 and 5 show the protein bands formed after incubation for 30 minutes at room temperature of a reaction containing 0.9 mg protein, 2 mM NAD^+, 14 μM of 20 bp duplex DNA in a buffer containing 30 mM HEPES, pH 7.5; 100 mM potassium acetate, 4 mM $MgCl_2$. PARylation of the protein is visible as a smeared band.

Calibration of magnetic tweezers

In this study, we employed two magnet configurations using pairs of 5mmx5mmx5mm cube N50 magnets (Supermagnetman). The first configuration had a 2mm gap between the magnets and the magnets were mounted on a rotating motor to enable DNA supercoiling together with a piezo controller for adjusting height above the flow cell. The second configuration had a 0.3 mm gap and was used with a fast piezo actuator. To calibrate the first magnet configuration, we used an 8 kbp length dsDNA molecule and calculated the applied force using $F = \frac{kTl}{\langle x^2 \rangle}$ where l is the tether length, k is the Boltzmann constant and $\langle x^2 \rangle$ is the mean-squared displacement parallel to the magnetic field lines (6). We fit the relationship between force and magnet height with a single exponential in the range of approximately 0.2 to 10 pN. The mean value from N=12 magnetic beads was given by $F = 27.8e^{-0.59z}$ where F is the force in pN and z is the distance in mm between the top cover slip and the magnets.

It is well-known that there is bead-to-bead variation in force in magnetic tweezers due to the variation in magnetic content of each bead. Dynabeads, which are used in this study, are known to have a standard deviation in force on the order of 10% (7). The 2 mm gap configuration was used for Figures 1C, 1D and 3. In these experiments a small variation in force on this order does not affect the interpretation of the experiment and therefore we used the average value given by the magnet law above (ie not taking into account bead-to-bead variation). For all other experiments we used the magnet configuration with a 0.3 mm magnet gap. For this configuration, we calibrated each bead individually by the following method. Firstly, we determined an overall average magnet law from many beads by measuring two known force transitions in a DNA hairpin structure. We used a short 75bp hairpin with 50% GC content which undergoes a single unfolding step at 14 pN (1, 8). We then measured the magnet height of unfolding of this hairpin together with the magnet height of the B-S transition. The position of the B-S transition has been accurately measured in a number of experiments with a midpoint at 65 pN for the ionic strength used (20mM Tris-HCl pH 7.5, 150 mM NaCl) (9–11). We measured the hairpin transition and B-S transition for 182 separate magnetic beads across multiple fields of view. The measurement of these two known force calibration points enables us to fit a single exponential $F = Ae^{-Bz}$ and calculate an average exponential decay of $\mathrm{B} = 1.05 \pm 0.04$ (mean±s.d.). This compares well with a literature report which used the same magnet configuration and found a magnet law with a decay factor of $\mathrm{B} = 1.07 \pm 0.05$ (12). To account for the bead-to-bead variation in each experiment we then measured the B-S transition for each bead after formation of the break with NruI so that the DNA was torsionally unconstrained (and before adding PARP proteins to the experiment). This was used to calculate the prefactor of the exponential for each bead independently (using the mean value of B = 1.05 for the decay constant). We used the method described above rather than fluctuation analysis since fluctuation measurements are complicated by a variety of factors at high force including the finite camera acquisition time and any force induced unbinding from the surface (13).

Name	Sequence
Biotin handle – primer1	5′-AATCTGCTGCAATGCCACAG-3′
Biotin handle – primer2	5′-GATGGTCTCATGCCGCAACAGCACAACCCAAACTG-3′
Spacer 1 – primer1	5′-GATGGTCTCAGGCAGCAACAGCACAACCCAAACTG-3′
Spacer 1 – primer2	5′-GATGGTCTCAATTGAATCTGCTGCAATGCCACAG-3′
Spacer 2 – primer1	5′-GATGGTCTCATGGGCCGTGGAATGAACAATGGAAGTC-3′
Spacer 2 – primer2	5′-GATGGTCTCAGCTCCTGTCAGGTGGCTCAATCTCTTC-3′
Digoxigenin handle – primer1	5′-GATGGTCTCAGAGCCCGTGGAATGAACAATGGAAGTC-3′
Digoxigenin handle – primer2	5′-CTGTCAGGTGGCTCAATCTCTTC-3′

Table S1. Primers used for PCR amplification of spacers and handles. The red letters indicate the overhang formed after digestion with BsaI. The purple letters indicate the BsaI recognition sequence.

Name	Sequence
3'-4bp oligo1	5′-PhosAGGTCGGCTCGCG-3′
3'-4bp oligo2	5′-AGCCGACCTAG-3′
5'-4bp oligo1	5′-PhosAGGTCGGCTCGCG-3′
5'-4bp oligo2	5′-CGCGCGCGAGCCGACCTAG-3′

Table S2. Oligonucleotide sequences used for generation of 3'-4bp and 5'-4bp overhangs. Oligo1 and oligo2 of each design (as above) were annealed to form a duplex. After incubation with DrdI, two overhangs with sequence of 5'-CT-3' are formed (the complementary sequence of the oligo is indicated in green). The desired duplex was then added to the flow cell together with T4 DNA ligase while applying sufficient force to hold the ends apart. The ligase was then washed out before adding T4 polynucleotide kinase to phosphorylate the 5' ends.

1. N. A. W. Bell, J. E. Molloy, Efficient golden gate assembly of DNA constructs for single molecule force spectroscopy and imaging. *Nucleic Acids Res.* **50**, e77 (2022).
2. N. Sherck, *et al.*, End-to-End Distance Probability Distributions of Dilute Poly(ethylene oxide) in Aqueous Solution. *J. Am. Chem. Soc.* **142**, 19631–19641 (2020).
3. C. Bouchiat, *et al.*, Estimating the Persistence Length of a Worm-Like Chain Molecule Biophysical. *Biophys. J.* **76**, 409–413 (1999).
4. P. L. Hansen, J. A. Cohen, R. Podgornik, V. A. Parsegian, Osmotic properties of poly(ethylene glycols): Quantitative features of brush and bulk scaling laws. *Biophys. J.* **84**, 350–355 (2003).
5. M. J. Shon, S. H. Rah, T. Y. Yoon, Submicrometer elasticity of double-stranded DNA revealed by precision force-extension measurements with magnetic tweezers. *Sci. Adv.* **5**, eaav1697 (2019).
6. E. Ostrofet, F. S. Papini, D. Dulin, Correction-free force calibration for magnetic tweezers

experiments. *Sci. Rep.* **8**, 15920 (2018).

7. H. Chen, *et al.*, Improved high-force magnetic tweezers for stretching and refolding of proteins and short DNA. *Biophys. J.* **100**, 517–523 (2011).

8. M. T. Woodside, *et al.*, Nanomechanical measurements of the sequence-dependent folding landscapes of single nucleic acid hairpins. *Proc. Natl. Acad. Sci. U. S. A.* **103**, 6190–6195 (2006).

9. S. B. Smith, Y. Cui, C. Bustamante, Overstretching B-DNA: The elastic response of individual double-stranded and single-stranded DNA molecules. *Science* **271**, 795–799 (1996).

10. H. Fu, H. Chen, C. G. Koh, C. T. Lim, Effects of magnesium salt concentrations on B-DNA overstretching transition. *Eur. Phys. J. E* **29**, 45–49 (2009).

11. J. R. Wenner, M. C. Williams, I. Rouzina, V. A. Bloomfield, Salt dependence of the elasticity and overstretching transition of single DNA molecules. *Biophys. J.* **82**, 3160–3169 (2002).

12. I. Popa, *et al.*, A HaloTag Anchored Ruler for Week-Long Studies of Protein Dynamics. *J. Am. Chem. Soc.* **138**, 10546–10553 (2016).

13. P. Daldrop, H. Brutzer, A. Huhle, D. J. Kauert, R. Seidel, Extending the range for force calibration in magnetic tweezers. *Biophys. J.* **108**, 2550–2561 (2015).
